# Rapid Characterization of Complex Killer Cell Immunoglobulin-Like Receptor (KIR) Regions Using Cas9 Enrichment and Nanopore Sequencing

**DOI:** 10.3389/fimmu.2021.722181

**Published:** 2021-09-14

**Authors:** Jesse Bruijnesteijn, Marit van der Wiel, Natasja G. de Groot, Ronald E. Bontrop

**Affiliations:** ^1^Comparative Genetics and Refinement, Biomedical Primate Research Centre, Rijswijk, Netherlands; ^2^Theoretical Biology and Bioinformatics, Utrecht University, Utrecht, Netherlands

**Keywords:** recombination, KIR, nanopore sequencing, targeted enrichment, Cas9

## Abstract

Long-read sequencing approaches have considerably improved the quality and contiguity of genome assemblies. Such platforms bear the potential to resolve even extremely complex regions, such as multigenic immune families and repetitive stretches of DNA. Deep sequencing coverage, however, is required to overcome low nucleotide accuracy, especially in regions with high homopolymer density, copy number variation, and sequence similarity, such as the *MHC* and *KIR* gene clusters of the immune system. Therefore, we have adapted a targeted enrichment protocol in combination with long-read sequencing to efficiently annotate complex *KIR* gene regions. Using Cas9 endonuclease activity, segments of the *KIR* gene cluster were enriched and sequenced on an Oxford Nanopore Technologies platform. This provided sufficient coverage to accurately resolve and phase highly complex *KIR* haplotypes. Our strategy eliminates PCR-induced amplification errors, facilitates rapid characterization of large and complex multigenic regions, including its epigenetic footprint, and is applicable in multiple species, even in the absence of a reference genome.

## Introduction

Repetitive regions are difficult to resolve using short-read sequencing approaches, and often remain registered for years as incomplete gaps in draft genomes (1–7). These complex stretches often involve transposable elements, microsatellites, and multi-copy gene clusters, the latter of which is represented by multiple gene families that encode essential components of the immune system (8, 9). For example, the major histocompatibility complex (MHC) genes, known in humans as human leukocyte antigens (HLA) genes, are considered the most polymorphic gene cluster. The *MHC* genes co-evolved with their receptors of the killer cell immunoglobulin-like receptor (KIR) gene family, which also features striking levels of complexity (10, 11).

The fundamental limitations to characterizing these complex regions by short-read sequencing strategies are potentially overcome by third-generation techniques that generate high yields of long reads (12, 13). Oxford Nanopore sequencing may produce reads far above 100 kb by recording changes in the electrical current as nucleotides pass through synthetic nanopores. The data quality and throughput of nanopore sequencing is improving rapidly, and has allowed the *de novo* assembly of multiple human genomes (14–16). These genome assemblies contiguously span multigenic clusters, such as the *MHC* and *KIR* gene regions, but correct annotation is hampered by the relatively low coverage, which precludes at this stage an accurate allele level resolution. Considering the important role of different multi-copy gene families in health and disease, a cost-efficient and high-resolution characterization approach regarding these types of regions is urgent.

Instead of whole genome sequencing, specific genes and regions might be enriched during library preparation. For instance, the *MHC class II DRB* gene region was enriched by long-ranged PCR, and characterized using a hybrid sequencing approach that combined Illumina and Oxford Nanopore platforms (17). Amplification steps, however, might introduce nucleotide errors during synthesis and, in addition, erase all epigenetic footprints. An amplification-free enrichment technique involves Cas9-mediated targeting of chromosome segments and nanopore sequencing (18–22). The Cas9 endonuclease activity may specifically excise genomic regions of interest that are subsequently ligated to nanopore adapters. This allows the direct sequencing of genomic segments while avoiding error prone DNA synthesis and maintaining epigenetic modifications. Efficient and specific enrichment using this approach has been demonstrated for single genes, including several cancer-related fusion genes (21), but an application for multigenic regions is absent in the literature.

In this study, we adapted the Cas9-mediated enrichment potential to resolve complex immune regions and validated this approach by the targeted characterization of *KIR* gene clusters in two different primate species. We focused on the *KIR* region in humans, which has been thoroughly characterized at the genomic level, and is important, for instance, in AIDS susceptibility and transplantation biology (23, 24). Rhesus macaques, however, represent a frequently used species in preclinical health research concerning, for example, COVID-19 and AIDS (25–27), but the physical location of the *KIR* genes is poorly understood. The *KIR* receptor family is involved in the regulation of NK cell activity and comprises activating and inhibitory members that may recognize particular epitopes on MHC class I molecules. A comprehensive nomenclature system distinguishes the variety of KIR receptors, and reflects the number of extracellular domains (KIR1D, KIR2D, KIR3D) and the length of the cytoplasmic tail (long, L; short, S) (28, 29). Subsequent numbering defines structurally similar but phylogenetically distinct genes (e.g., *KIR2DL1*), whereas three additional digits distinguish allotypes (e.g., KIR2DL1*001). In humans, a total of 17 *KIR* genes are defined, 1110 alleles of which are documented (IPD-KIR, release 2.9.0) (30).

The *KIR* gene repertoire is shaped by abundant tandem duplications, deletions, and chromosomal recombination events, and exceeds the plasticity of the *MHC* gene cluster (11, 31, 32). The *KIR* genes are 10 to 15 kb long and are arranged in a head-to-tail manner, separated by intergenic regions of approximately 2 kb. Sequence similarity characterizes the genetic cluster, with any two *KIR* genes sharing 80%–90% homology, and allelic variants of a certain gene tend to be over 98% similar. The KIR haplotypes, defined as a segregating unit of genes located on a single chromosome, distinguish a centromeric and telomeric segment, which display diverse configurations and extensive copy number variation. The *KIR* genes display a variegated expression pattern, which is modulated by methylation of the promotor regions (33). For this study, we resolved six human *KIR* haplotypes, derived from three randomly selected human donors, at an allele-level resolution and additionally determined their methylation profiles.

The continuous evolution of the *KIR* gene system is reflected by the genomic diversification at a species, population, and individual level. To validate our concept, we enriched and assembled KIR haplotypes in rhesus macaques. So far, only two completely sequenced rhesus macaque *KIR* haplotypes have been documented, but previous transcriptome and segregation studies indicate extensive variation (34–38). Annotation of this complex immune region is a difficult enterprise, which is reflected by a poorly annotated *KIR* region in the rhesus macaque reference genome (Mmul_10) (39). Our genomic characterization of rhesus macaque *KIR* haplotypes demonstrated the rapid construction of complex multigenic haplotypes, even in the absence of reference sequences. Hence, adaption of this technique allows a speedy and cost-efficient characterization of other immune regions, from which whole genome assemblies and clinical implications might benefit.

## Materials and Methods

### Cells and Genomic DNA Extraction

Human buffy coat samples from healthy donors were obtained from the Dutch blood bank (Sanquin, the Netherlands). Informed consent was obtained from all participants. Rhesus macaques with a characterized KIR transcriptome were selected from the self-sustaining colony housed at the Biomedical Primate Research Centre (BPRC) (28, 35). Heparin whole blood samples from these animals were obtained during annual health checks. PBMCs were isolated from human buffy coats and rhesus macaque heparin samples.

High-molecular-weight (HMW) gDNA was isolated from human and rhesus macaque PBMC samples (± 7 x 10^6^ cells), using the Circulomics Nanobind CBB Big DNA Kit (Circulomics, NB-900-001-01) and following the manufacturer’s instructions. The concentration and purity of the gDNA samples were determined using a Nanodrop and a Qubit platform. The (HMW) gDNA fragment length was determined by pulsed field gel electrophoresis (PFGE) in reference to a lambda PFGE ladder.

### Designing Guiding crRNAs and Constructing RNPs

For both humans and macaques, sets of generic and specific CRISPR RNAs (crRNA) were designed within the *KIR* gene cluster and the flanking *LILR* and *FCaR* genes by using Benchling, a freely available online software tool (40). The crRNAs have a guiding length of 20 bp, and are designed in front of a protospacer adjacent motif (PAM) sequence “NGG”, in which “N” represents any nucleotide base. The software tool provides on- and off-target scores for specific crRNAs. The on-target scores are based on optimized calculations from Doench and colleagues, with the higher score indicating the better crRNA target binding (41). The off-target scores reflect the specificity of the crRNAs, and were based on different builds of the human (NCBI36, GRCh37, GRCh38) and macaque (MMUL_1, Mmul_8.0.1) reference genomes (42). CRISPR RNAs with high off-target scores were considered specific for one *KIR* gene. Relatively low off-target scores (ranging from 3 to 60) were recorded for crRNAs that potentially target multiple *KIR* genes and were included in the panel as putative generic crRNAs. In total, 54 and 45 custom crRNAs were selected to enrich the human and macaque *KIR* gene cluster, respectively (**Supplementary Tables 1**, **2**) (IDT, custom Alt-R^®^ CRISPR-Cas9 crRNA).

Sets of different crRNAs were pooled based on the different strand-directed orientations (sense versus anti-sense) to avoid cleavage and the sequencing of unintended short on-target fragments. The pools were defined to generate DNA fragments that comprise *KIR* genes from exon 1 to exon 9, or fragments that connect neighboring *KIR* genes (**Supplementary Tables 1** and **2**). The pooled crRNAs were mixed with trans-activating crRNAs (tracrRNA) (IDT, #1072534) in a 1:1 ratio and further diluted in Duplex Buffer to a final concentration of 10 μM. The crRNAs and tracrRNA were annealed by heating the duplex solution for 5 min at 95°C, followed by cooling to room temperature (RT) on a benchtop. To subsequently construct the Cas9 ribonucleoprotein particles (RNPs), the crRNA-tracrRNA duplexes were assembled with HiFi Cas9 endonuclease (IDT, #1081060) in 1x NEB CutSmart Buffer (NEB, #B7204S) at a total volume of 30 μl by incubating the solution for 30 min at RT. The Cas9 RNPs were stored until use at 4°C for up to a week.

### Cas9-Mediated Target Enrichment and Oxford Nanopore Sequencing

**Figure 1:**
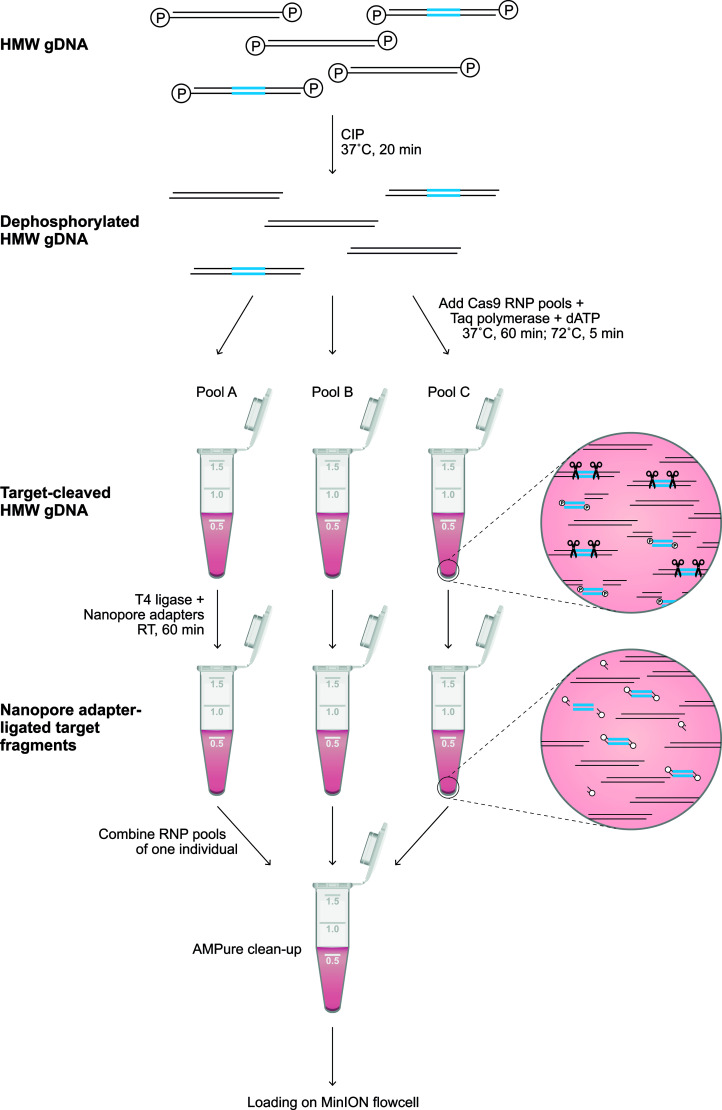
Library preparation of the targeted enrichment protocol. A schematic overview of the library preparation to enrich a target region (blue). Freshly isolated HMW genomic DNA was dephosphorylated by incubation with CIP for 20 min at 37°C. Subsequently, selected pools of RNPs (**Supplementary Tables 1, 2**) were added to aliquots of the dephosphorylated gDNA, and incubated for 60 min at 37°C in the presence of Taq polymerase and dATP, followed by incubation for 5 min at 72°C. The available phosphate groups at the terminus of the targeted region were ligated to Nanopore adaptors during incubation with T4 ligase for 60 min at room temperature (RT). The adapter-ligated aliquots were pooled, followed by clean-up with 0.3X AMPure beads. The eluted sample was loaded on a flowcell for Nanopore sequencing.

Throughout the protocol, unintended fragmentation of gDNA will decrease the capturing efficiency and enrich off-target fragments. Therefore, samples should be handled with care and processed with wide-bore pipette tips. Input gDNA (5-10 μg) was resuspended in 10x NEB CutSmart Buffer (8:1) and dephosphorylated by incubation with Quick calf intestinal phosphatase (CIP) (NEB, # M0525S) at 37°C for 20 min, followed by heating at 80°C for 2 min to deactivate the enzyme (**Figure 1**). After the sample returned to room temperature, the dephosphorylated gDNA (30 μl) was gently mixed with a Cas9 RNP pool (10 μl), 10 mM dATP (1 μl), and Taq polymerase (1 μl), followed by incubation at 37°C for 60 min, then 72°C for 5 min, and hold at 4°C. Ligation buffer (20 μl) and sequencing adaptors (5 μl) from the Ligation Sequencing Kit (ONT, #LSK109) and Quick T4 DNA Ligase (10 μl) (NEB, M2200S) were added to the cleaved and dA-tailed gDNA sample, followed by an incubation of 60 min at RT. Adapter-ligated samples from the same individual, which were treated with different crRNA sets, were pooled, and diluted (1:1) in TE buffer. The excess of adaptors and short DNA fragments were removed using 0.3x AMPure XP Beads (Beckman Coulter, #A63881), which were washed twice on a magnetic rack with Long Fragment Buffer (ONT, #LSK109). The beads were eluted in 15 μl Elution Buffer (ONT, #LSK109). A sequencing library was prepared by adding 37.5 μl Sequencing Buffer and 25.5 μl Loading Beads (ONT, #LSK109) to the processed DNA sample. Eluted samples were sequenced on a R9.4.1 flowcell using an Oxford Nanopore MinION device. Prior to sequencing, the flowcells were primed according to the manufacturer’s instructions using the Flow Cell Priming Expansion Pack (ONT, #LSK109). After 24 hours of sequencing, flowcells with over 500 active pores remaining were washed and reloaded with a second enriched library of the same gDNA sample according to the manufacturer’s instructions using the Flow Cell Wash Kit (ONT, #EXP-WSH003).

### Sequence Data Analysis

Base calling and read quality assessment (min qscore 7) were performed using Guppy V3.4.1 software on a Linux platform that utilized a GeForce RTX 2080 Ti graphics processing unit (GPU). Base called reads were imported into Geneious Prime software (v.2020.1.2) for further analysis. Exon libraries, including sequences of exons 3, 4, and 7 from the human and rhesus macaque KIR databases (IPD-KIR, release 2.9.0; IPD-NHKIR, release 1.3.0.0), were used as references to map the Nanopore reads into contigs based on similarity. Each on-target contig resembled a specific *KIR* gene or a pair of neighboring *KIR* genes.

For each human individual, the haplotype configurations (e.g., cA01-tA01) could be largely deduced from the read contigs generated by exon library mapping. On the basis of this knowledge, the reads of a single flowcell were re-mapped to the complete human reference genome (HG38), complemented with the particular *KIR* haplotype configuration references, using minimap2 (version 2.17). Consensus sequences that covered *KIR* genes from start to end, or that comprised segments of neighboring *KIR* genes, were generated from the alignments based on 65% nucleotide similarity. The consensus accuracy was determined by comparison to the reference haplotype. The on-target coverage was resolved for each individual by mapping all reads with a read length above 7,000 bp to the human reference genome (HG38) using minimap2 in Geneious software. The enrichment factor was determined as the ratio of on- and off-target mean coverage, and reflects the enrichment efficiency.

For each rhesus macaque sample, reads from the on-target exon contigs were re-mapped to the contig consensus sequence using minimap2. Additional re-mapping steps were performed using the generated consensus sequences to optimize accuracy and diminish homopolymer errors. To further optimize these consensus sequences, shorter on-target reads (< 7,000 bp) were included for consensus calculations. The eventual consensus sequences were generated based on 65% nucleotide similarity. The consensus accuracy was estimated by the alignment with exon references available from previous KIR transcriptome studies (IPD-NHKIR, release 1.3.0.0). On-target coverage was defined by mapping all reads with a length of 7,000 bp or more to the rhesus macaque reference genome (Mmul_10), which was complemented with the appropriate assembled *KIR* haplotypes, using minimap2 in Geneious software.

### DNA Modification Profiles

Raw signal data including information on DNA modifications, such as methylation, were processed using Guppy V4.0.12 software with the dna_r9.4.1_450bps_modbases_dam-dcm-cpg_hac.cfg configuration and fast5_out flag. Multi-read fast5 files were converted to single-read files using multi_to_single_fast5 provided by ONT. The base called reads were mapped to a reference genome using minimap2, and subsequently called for modifications using Nanopolish (43). The methylation likelihood and frequencies were visualized by Methplotlib (44), using an annotated reference genome. When the reference genome did not contain the appropriate *KIR* genes, like Mmul_10 for all macaque individuals, methylation calls were annotated using a modified reference genome, which was complemented with the newly assembled haplotypes as artificial chromosomes.

## Results

### A ‘Tiling’ Approach to Enrich Complex Immune Clusters Without Amplification

The characterization of large and repetitive immune regions requires the generation and sequencing of genomic DNA (gDNA) fragments that share overlapping segments. Allelic variation in these overlaps allows the phasing of haplotypes. To achieve this goal, dephosphorylated high molecular weight (HMW) gDNA needs to be cleaved, using sets of CRISPR RNAs (crRNA) in complex with Cas9 endonuclease (**Figure 1**). These crRNAs are designed to target conserved stretches that are shared by members of a multigenic family. This approach will allow generic enrichment. Only at the terminus of the cleaved target sites is a phosphate group available, which is utilized for dA-tailing and subsequent ligation to Nanopore sequencing adaptors. This ‘tiling’ approach facilitates the selective enrichment of large overlapping DNA-segments, and allows the subsequent sequencing of polymorphic and multigenic immune regions without the need for amplification.

### Enrichment of Complex KIR *Regions*

**Figure 2:**
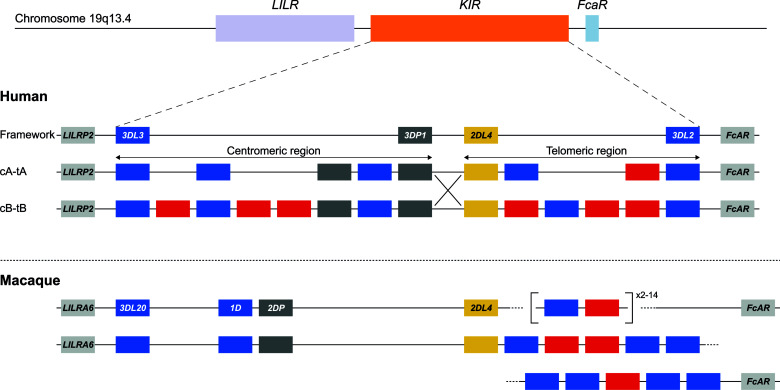
KIR haplotype configurations in humans and rhesus macaques. The *KIR* gene family maps between the *LILR* cluster and the *FcAR* gene. In humans, four genes are considered to reflect a framework reference haplotype, with *KIR3DL3* and *KIR3DP1* defining the centromeric region (c), and *KIR2DL4* and *KIR3DL2* bounding the telomeric region (t). In the human population, both the c and t regions may feature different gene contents. The red and blue boxes depict activating or inhibitory receptors, respectively. Pseudogenes have been indicated as well (grey). The only homologous gene shared in humans and macaques is *KIR2DL4* (yellow). Human *KIR* haplotypes are categorized based on a more inhibitory (group A) or activating (group B) gene profile, and reflect different segment configurations (e.g., cA-tA and cB-tB) (31). Chromosomal recombination events might shuffle haplotype segments (e.g., cA-tB defined by the large X). In rhesus macaques, the framework is represented by *KIR3DL20* at the centromeric region. The telomeric region is characterized by highly variable gene expansions and contractions. In contrast to the situation for humans, physical maps of rhesus macaque KIR haplotypes are virtually absent from the literature. For that reason, the lower panel represents a hypothetical configuration as the proposed situation at the start of this study.

**Figure 3:**
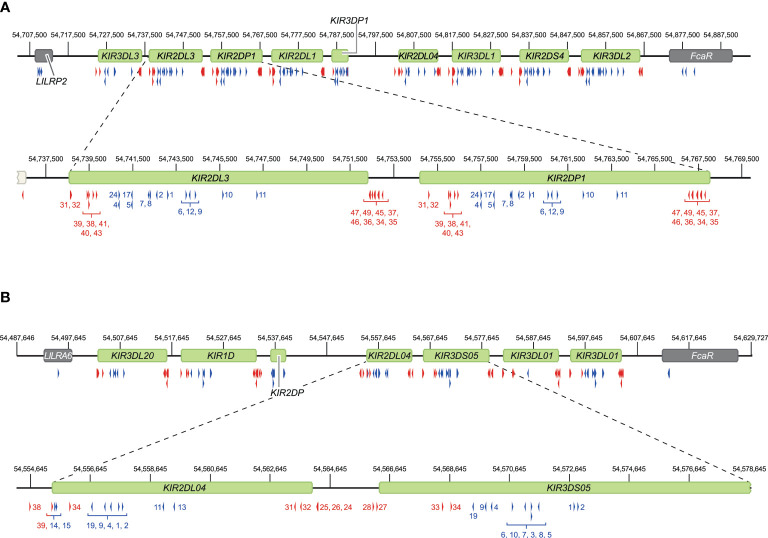
Overview of crRNA sets to target human and rhesus macaque KIR haplotypes. Sets of crRNAs were designed to guide the Cas9 nuclease enzyme to the *KIR* gene cluster in humans **(A)** and rhesus macaques **(B)**. The cluster location at the reference genomes, HG38 and Mmul_10, is provided. Representative regions are enlarged for a more specific indication of the cut sites. Blue and red arrows reflect crRNAs that excise gene to gene fragments and complete gene fragments, respectively, and are indicated with numbers corresponding to **Supplementary Tables 1, 2**.

To validate our approach, the *KIR* gene regions in humans and rhesus macaques were enriched and characterized (**Figure 2**). The nature of *KIR* gene complexity required the design of species-specific sets of crRNAs to enrich complete genomic clusters (**Figure 3** and **Supplementary Tables 1**, **2**). In humans, the presence of four framework genes with distinctive physical locations marks the centromeric (*KIR3DL3* and *KIR3DP1*) and telomeric (*KIR2DL4* and *KIR3DL2*) haplotype segments (**Figure 2**). Expansion and contraction of both regions resulted in haplotypes that contain 9 to 14 *KIR* genes, including two pseudogenes (*KIR2DP1* and *KIR3DP1*). Important to note, however, is that some genes that are present on a given *KIR* haplotype may be absent from another. Human *KIR* haplotypes can be roughly categorized based on their gene content, into those with more an inhibitory (group A) or an activating (group B) gene profile (45). Recombination events, possibly owing to the high transposon density, might rearrange haplotype organizations (**Figure 2**). To determine the high content variability of this genetic cluster, 35 generic crRNAs were designed to target the differential presence of human *KIR* genes that may be encountered on a haplotype, whereas 12 crRNA were specific for one particular framework gene (**Figure 3A** and **Supplementary Table 1**). In addition, seven crRNA were included to target the genes that flank the *KIR* gene cluster (*LILR* and *FcAR*), in order to define both ends of the *KIR* haplotype.

The *KIR* haplotypes in rhesus macaques display even more content diversity, with 4 to 17 KIR transcripts encoded as defined by segregation studies (34, 35). The only framework gene present on all haplotypes is *KIR3DL20*, which marks the centromeric region (**Figure 2**). Only two other *KIR* genes are differentially located within this haplotype segment (*KIR1D* and *KIR2DP*). The only macaque KIR ortholog that is shared with humans, *KIR2DL04*, is present on approximately 80% of the telomeric haplotype segments. This haplotype region is further characterized by a differential number of *KIR* genes in highly diverse frequencies. To enrich the macaque *KIR* cluster, we designed 24 generic crRNAs that target the variable gene tandem, and complemented those with crRNAs specific for *KIR1D*, *KIR2DL04*, and *KIR3DL20*, and the flanking genes, *LILRA6* and *FcAR* (**Figure 3B** and **Supplementary Table 2**).

### Assembly of Human KIR Haplotypes

For this communication, the human target region was defined at the start of exon 5 of *LILRP2* to the end of exon 1 of *FcAR*, thereby comprising the complete *KIR* haplotype (**Figure 2**). On the human reference genome (HG38), this target region spans approximately 161 kb and contains nine *KIR* genes, one of which encodes an activating KIR (group A haplotype). To account for the variability of *KIR* haplotype configurations, four additional reference sequences, which were assembled by Fosmid sequencing and reflected different *KIR* genotypes, were included in our panel (46). In total, five reference *KIR* regions were used to assemble captured reads and to determine sequence accuracy.

**Figure 4:**
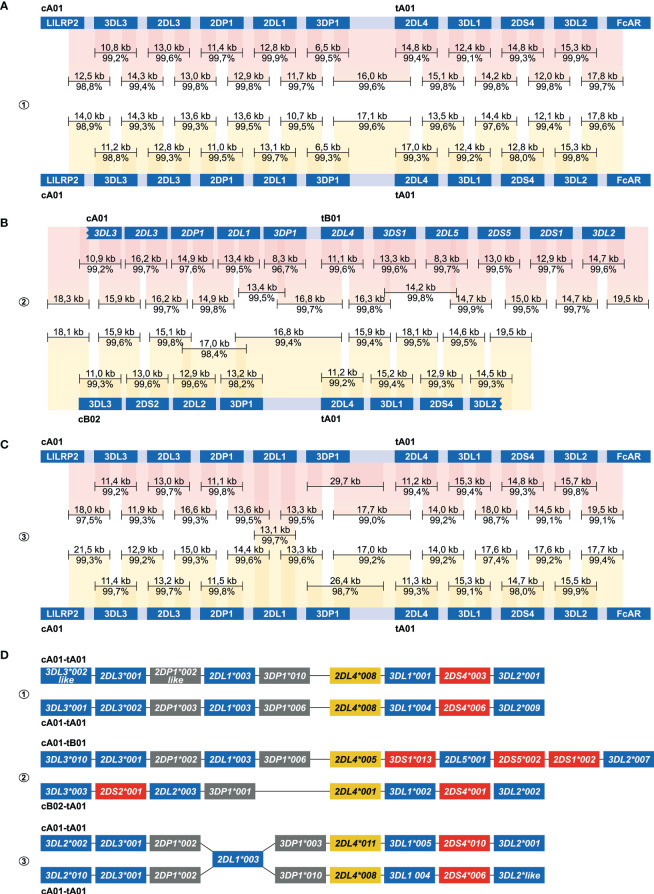
Enrichment and phasing of human KIR haplotypes. Human donors 1, 2, and 3 were randomly selected, and displayed one heterozygous (#2) and two homozygous (#1, #3) *KIR* haplotype configurations. Consensus sequences (black bars) that covered KIR genes from start to end, or that comprised segments from neighbouring *KIR* genes, were mapped to corresponding haplotype references derived from the human reference genome (HG38) or from previous Fosmid sequencing studies (46). The consensus sequence length and accuracy are indicated. Colored columns indicate the mapped regions, in which a darker color illustrates overlapping consensus sequences. KIR haplotypes were completely phased in two individuals **(A, B)**. The third individual shared an identical *KIR2DL1* gene on both haplotypes, which located an extended SNP desert (25 kb; **Figure S2**) **(C)**. *KIR* haplotypes of this individual were phased at all genes at an allele level resolution, except for the *KIR2DL1* gene. Four differential *KIR* region configurations from three randomly selected donors were resolved. Based on allelic polymorphism, each of these haplotypes appears to be unique **(D)**. In addition, three new variants of documented alleles were defined, which are indicated with “like”.

For each of the three randomly selected human individuals, two Nanopore flowcells were loaded with HMW gDNA samples that were enriched either for fragments that comprise a complete *KIR* gene or fragments that connect and distinguish neighboring *KIR* genes. An average of 1.26 million Nanopore reads were yielded per flowcell (**Table 1**). Of these reads, 4.2% to 19.3% had a length of 7,000 bp or longer, which is required to connect and distinguish neighboring *KIR* genes. The percentage of size-selected reads that mapped to the target region ranged from 1.6% to 2.5% (± 4,015 reads), which provided a median coverage ranging from 269 to 323X. The enrichment factor, which reflects the efficiency of the targeted enrichment, ranged from 215 to 394X. The length of the consensus sequences that were generated from the on-target reads ranged from 6.5 to 29.7 kb, and their assembly covered complete reference *KIR* haplotype configurations (**Figure 4C**). The consensus accuracy compared to the reference sequences ranged from 96.7% to 99.9%.

**Table 1:** Overview of the total reads, read length, on-target hits, and the coverage of the target region in human samples.

	Gene fragments (flowcell 1)		
	#1	#2	#3
Total # reads	789705	1175600	1413919
Total # reads > 7.000 bp	57830	49535	59575
Percentage reads > 7.000 bp	7.3	4.2	4.2
Average read length (bp)	2603	2114	2092
Average read length (bp) of reads > 7.000 bp	13410	10895	13317
	**Gene to gene fragments (flowcell 2)**		
	**#1**	**#2**	**#3**
Total # reads	1060000	1078502	2012903
Total # reads > 7.000 bp	204797	152372	108688
Percentage reads > 7.000 bp (%)	19.3	14.1	5.4
Average read length (bp)	5062	3728	2021
Average read length (bp) of reads > 7.000 bp	17206	13327	13455
	**Total reads (flowcells 1 and 2)**		
	**#1**	**#2**	**#3**
On-target reads (> 7.000 bp reads)	4470	3308	4268
Percentage on-target reads	1.7	1.6	2.5
Mean coverage target region	323	269	315
Enrichment factor	215	299	394
			

The centromeric and telomeric regions were completely assembled and grouped into four different segment configurations (cA01, cB02, tA01, tB01). Phasing of complete *KIR* haplotypes was achieved in two individuals, including a homozygous haplotype configuration using allele level resolution (**Figures 4A, B, D**). This result demonstrates the resolution power of our approach. For another homozygous setting, *KIR* haplotypes were phased for all genes, except for *KIR2DL1* (**Figure 4C**). This gene was identical on both haplotypes, and defined a SNP desert (25 kb) that hampered complete phasing (**Figure S1**). The largest completely assembled and phased haplotype comprised 11 *KIR* genes and covered a total of approximately 176 kb (**Figure 4B**).

### Assembly of Rhesus Macaque KIR Haplotypes Without Reference Genome

The successful deciphering of highly variable rhesus macaque *KIR* haplotypes validated our enrichment and characterization approach further, even in the absence of genomic reference sequences. The haplotype content was initially determined by previous transcriptome and segregation studies (34, 35), but the physical location of *KIR* genes remained elusive. The target region was defined at the start of exon 7 in *LILRA6* to the end of exon 2 in *FcAR*, thereby comprising the complete rhesus macaque *KIR* cluster. This region covered 330 kb on the rhesus macaque reference genome (Mmul_10). Ironically, the *KIR* gene cluster is poorly assembled and annotated on this reference genome and is at present not suitable as a reference to assemble *KIR* haplotypes.

The rhesus macaque *KIR* cluster was enriched from samples of three animals. One or two flowcells were used to enrich fragments that comprised *KIR* genes from start to end, or connected neighboring *KIR* genes. A range of 2.5% to 36.3% of the total reads had a read-length of 7,000 bp or longer (**Table 2**). The percentage captured on-target reads ranged from 0.5% to 4.2% (**Table 2**). The mean coverage of the target region was determined by mapping all reads to the reference genome (Mmul_10), which was complemented with the assembled *KIR* haplotypes as artificial chromosomes and reached 26X to 91X. The enrichment factor ranged from 128X to 637X. Consensus sequences were generated, which displayed lengths ranging from 7.2 to 20.0 kb. The accuracy of the consensus sequences is estimated by a comparison with rhesus macaque exon sequences extracted from the non-human KIR Database (IPD-NHKIR, release 1.3.0.0), and reached 96.7% to 100% similarity at these coding regions.

**Table 2:** Overview of the total reads, read length, on-target hits, and the coverage of the target region in rhesus macaque samples.

	Gene fragments (flowcell 1)		
	#1	#2	#3
Total reads	252981	453649	2208000
Total reads > 7.000 bp	25062	54772	55490
Percentage reads > 7.000 bp	9.9	12.1	2.5
Average read length (bp)	3098	3375	1978
Average read length (bp) of reads > 7.000 bp	11258	11875	9319
	**Gene to gene fragments (flowcell 2)**		
	**#1**	**#2**	**-**
Total reads	48586	20956	–
Total reads > 7.000 bp	5179	7607	–
Percentage reads > 7.000 bp (%)	10.7	36.3	–
Average read length (bp)	2884	7110	–
Average read length (bp) of reads > 7.000 bp	12226	14789	–
	**Total reads (flowcells 1 and 2)**		
	**#1**	**#2**	**#3**
On-target reads (> 7.000 bp reads)	1070	1005	292
Percentage on-target reads	4.3	1.8	0.5
Mean coverage target region	60.8	91.8	26.8
Enrichment factor	637	404	128

*For #3, a single flowcell was used for all different crRNA pools, generating fragments containing a KIR gene from start to end, and fragments that span from one KIR gene to another.

**Figure 5:**
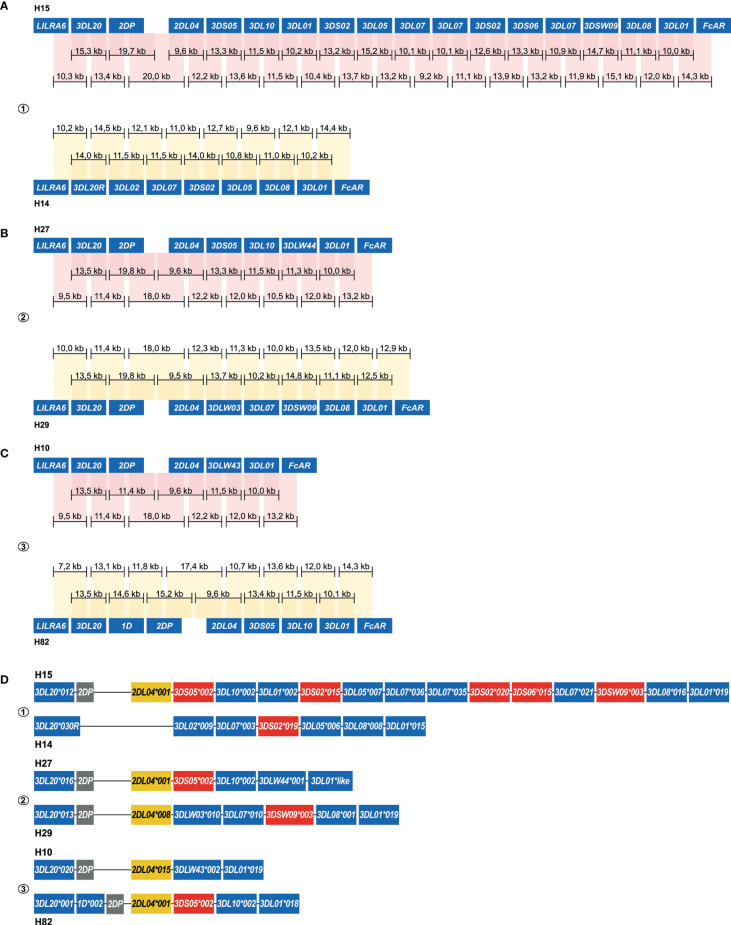
Enrichment and phasing of rhesus macaque *KIR* haplotypes. For rhesus macaque samples (#1, #2, and #3), the *KIR* gene content was determined by a combination of transcriptome and segregation studies (11, 32, 33). Our approach resulted in an accurate annotation of the complex *KIR* regions present in these animals. Consensus sequences (black bars) that covered *KIR* genes from exon 1 to exon 9, or that comprised segments from neighboring *KIR* genes, were assembled. The physical order of *KIR* genes at the haplotype was determined by the extensive overlaps and the alignment with exon sequences from the IPD-NHKIR. The consensus sequence lengths are indicated. Phasing was achieved for all haplotypes, and reflected six different configurations **(A–C)**. For each sample, haplotypes were sorted out at an allele level resolution **(D)**. One new allele was defined for *KIR3DL01* (H27), which is indicated with “like”.

An allele level resolution allowed the phasing of six rhesus macaque *KIR* haplotypes (**Figure 5**). Even with a single flowcell, sufficient coverage was reached to define haplotypes at an allele level resolution (**Figure 5C**). The largest haplotype contained 16 *KIR* genes and spanned 280 kb (H15). The different allelic copies of *KIR3DL01*, *KIR3DL07* and *KIR3DS01* that could be distinguished on this extended haplotype suggests its generation by multiple chromosomal recombination events. The shortest *KIR* haplotype encoded five *KIR* gene members (H10). A fusion gene, which consists of segments from two distinct *KIR* genes, and that are occasionally generated by chromosomal recombination events, was identified on H14 (KIR3DL20*030R) (**Figure 5A**). These recombined entities often remain undetected by current genotyping approaches.

### Methylation Profile of the Multigenic KIR Region

**Figure 6:**
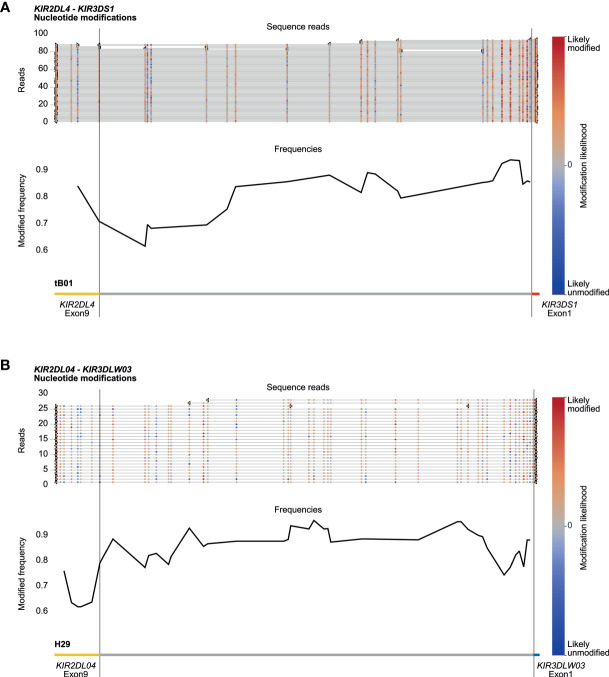
DNA modification profiles. DNA modification profiles are displayed for one human (sample #2) **(A)** and one rhesus macaque (sample #2) **(B)** *KIR* gene. The epigenetic predictions were calculated with up to 100 randomly selected reads per enriched *KIR* segment. Indicated are the modification likelihoods (red, high; blue, low), the modification frequency, which ranges from 0 to 1, and the annotation that is based on the (complemented) reference genomes (HG38 and Mmul_10). Plots were generated by Methplotlib (44). The *KIR* promotor region, which is approximately 300 bp in front of exon 1 (33), is highly modified for human *KIR3DS1* **(A)** and macaque *KIR3DLW03* **(B)**, with modification frequencies ranging from 85 to 95%. These two regions are representative for all other *KIR* gene regions studied in our human and rhesus macaque cohorts.

Amplification-free enrichment and nanopore sequencing allow the characterization of DNA modification profiles. *KIR* genes display a variegated expression on NK cells and subsets of T cells, which is tightly regulated by methylation of the promotor region (33). Therefore, we have investigated whether we could determine methylation signatures for the *KIR* gene region. The modification likelihood (low, blue; high, red) was defined for all positions on individual reads that mapped to the *KIR* cluster (**Figures 6A, B**, top part). Based on up to 100 reads, the frequency of modification was determined for different *KIR* gene promotor regions (**Figures 6A, B**, black line at the bottom part). A high modification likelihood and frequency was determined for the promotor region of all *KIR* genes in the human samples (**Figure 6A**). The modification frequency of the complete intergenic region ranged from 62% to 93% and seems to slightly increase in the proximal promotor adjacent to exon 1. The intergenic regions in the rhesus macaque *KIR* cluster also displayed high likelihood and frequency of modification (**Figure 6B**). However, the slight increase of modification frequency at the proximal promotor is not observed. Because all DNA samples were isolated from peripheral blood lymphocytes, the high methylation frequency is in line with scare expression of KIR on most prominent lymphocytes. The true resolution power of this approach would emerge using isolated cell populations or single-cell clones.

## Discussion

Most multigenic regions are subjected to complex evolutionary processes, in which species-specific gene duplications, deletions, and recombination events shape a genetic cluster. Immune gene families, such as KIR and MHC, diversified under selective pressure by a continuous arms race with pathogens, and individuals might benefit from extensive variation at the population level (47). The high gene content diversity and sequence similarity challenges rapid genomic characterization of these multigenic families.

We make available a rapid enrichment and characterization approach for complex immune regions, which overcomes many of the limitations of short-read or whole-genome sequencing. The Oxford Nanopore MinION sequencing device and flow cells are relatively inexpensive. This makes the described method widely accessible to laboratories without the need for dedicated sequencing facilities, which contrasts other long-read sequencing platforms, such as PacBio sequencing. Extensive diversity in gene content of multigene families was resolved by targeting long overlapping genomic fragments that covered genes from start to end, or that contained segments of neighboring genes. These enriched fragments display a median length of approximately 10 kb with outliers up to 25 kb, which is in line with the expected target fragments. Isolation of longer fragments might be achieved using Cas9-enrichment but was not required for this study design. Efficient targeting was based on using generic crRNAs, which yielded a relatively high median coverage at the region of interest and allowed the generation of accurate consensus sequences. Most inaccuracies involved homopolymers, here defined as a stretch of three or more identical bases, which simultaneously impact the current measured by the nanopore. The consensus sequence accuracy was, however, sufficient to define alleles and to phase complex *KIR* haplotypes. Only at large identical stretches in which overlapping fragments lack allelic variation might this technique hamper the phasing of haplotypes (**Figure S1**). These SNP deserts might be resolved by additional crRNAs that flank the identical stretch. In addition, the continuous improvement of library preparation chemistry, base-calling algorithms, and post-sequencing correction software might eventually increase the length of enriched fragments and the accuracy of nanopore sequencing.

The target coverage was sufficient for the characterization of *KIR* haplotypes at the allele level resolution, but the enrichment performance displayed some deviations in the different samples (**Tables 1**, **2**). The variance in total read numbers and read length is likely to be affected by flowcell variations, gDNA sample quality and short read clean-up efficiency during library preparation. Despite the deviations in read counts, the on-target coverage was sufficient, even when only a single flowcell was used (**Table 2**, #3 and **Figure 5C**). Further optimalization of the enrichment protocol, by designing additional crRNAs or by enhancing the Cas9 efficiency with improved chemistry, might provide higher on-target coverage. Samples might then be multiplexed on a single flow cell to further reduce the costs of KIR cluster characterization. Nonetheless, the current approach already ensured highly accurate and overlapping consensus sequences that cost-efficiently resolved complete human and macaque *KIR* haplotypes at an allele level resolution (**Figures 4D**, **5D**).

The KIR receptors display a selective and variegated expression on the cell surface of NK cells and subsets of T cells (48, 49). The majority of these cells only express a single *KIR* gene, whereas smaller fractions might have two to four KIR receptors on their cell surface. This selective expression pattern is most likely regulated by methylation of the promotor region, which is initiated during NK cell differentiation (50). The high modification frequency of the *KIR* promotor regions (**Figure 6**) indicates low levels of expression in the characterized samples (33). Our HMW DNA samples were isolated from whole blood, which includes only a small fraction of KIR-positive cells that might display hypomethylated promotor regions. Nevertheless, the epigenetic modification of members from highly complex multigenic families could be determined using our approach.

Resolving complete multigenic haplotypes and their epigenetic profiles at the allele level resolution might provide biological and diagnostic insights into the role of these complex systems in health and disease. This is thoroughly demonstrated for different combinations of KIR and HLA that might have an impact on human health, susceptibility or resistance to disease, or may affect graft survival success in transplantation biology (23, 24, 51). There is, however, a lack of consensus from these association studies. One of the causes that might explain these contradicting outcomes is the predominant strategy to characterize common structural motifs, such as centromeric and telomeric KIR haplotype segments, thereby simplifying the plasticity of these immune regions. Using our rapid enrichment and sequencing approach, more comprehensive associations might be defined based on completely defined haplotypes at the allele level resolution.

Members of multigenic families often encode components of essential immune responses and exhibit extensive copy number variation and allelic polymorphism. The continuous diversification and selection of these genes enables adaption to pathogens but might also generate candidates that enhance susceptibility to immune-related disorders. These adaptions might involve SNPs that are mapping in coding regions, whereas others are not, and may impact, for instance, gene (expression) regulation and alternative splicing potential. This enrichment technique fosters a cost-efficient and rapid strategy to characterize complex immune-receptor families at an allele level. Insights regarding complex immune clusters might provide a comprehensive perspective on biological interpretations, and lift SNP disease association studies from the allele to the haplotype/region level, in which all polymorphisms are considered.

## Data Availability Statement

All raw Nanopore files and processed sequencing data generated in this study have been submitted to the European Nucleotide Archive (ENA) (https://www.ebi.ac.uk/ena/browser/home) under accession number PRJEB43311.

## Author Contributions

JB and MW performed all practical work. JB wrote the manuscript. NG and REB supervised the project and edited the manuscript. All authors contributed to the article and approved the submitted version.

## Funding

This work was supported by the Biomedical Primate Research Centre.

## Conflict of Interest

The authors declare that the research was conducted in the absence of any commercial or financial relationships that could be construed as a potential conflict of interest.

The reviewer LW has declared a past collaboration with the author REB to the handling editor at the time of review.

## Publisher’s Note

All claims expressed in this article are solely those of the authors and do not necessarily represent those of their affiliated organizations, or those of the publisher, the editors and the reviewers. Any product that may be evaluated in this article, or claim that may be made by its manufacturer, is not guaranteed or endorsed by the publisher.

## Supplementary Material

Supplementary Figure 1 A SNP desert at the *KIR2DL1* gene. Human individual #3 shared an identical *KIR2DL1* allele at both haplotypes, which located a 25 kb SNP desert. The lack of reads that span the complete SNP desert hampered the phasing of *KIR2DL1*.
