# Supplementary figures and images for: Rapid Characterization of Complex Killer Cell Immunoglobulin-Like Receptor (KIR) Regions Using Cas9 Enrichment and Nanopore Sequencing
Source: Front Immunol. 2021 Sep 14;12:722181. doi: 10.3389/fimmu.2021.722181 (PMC8476923; doi:10.3389/fimmu.2021.722181)

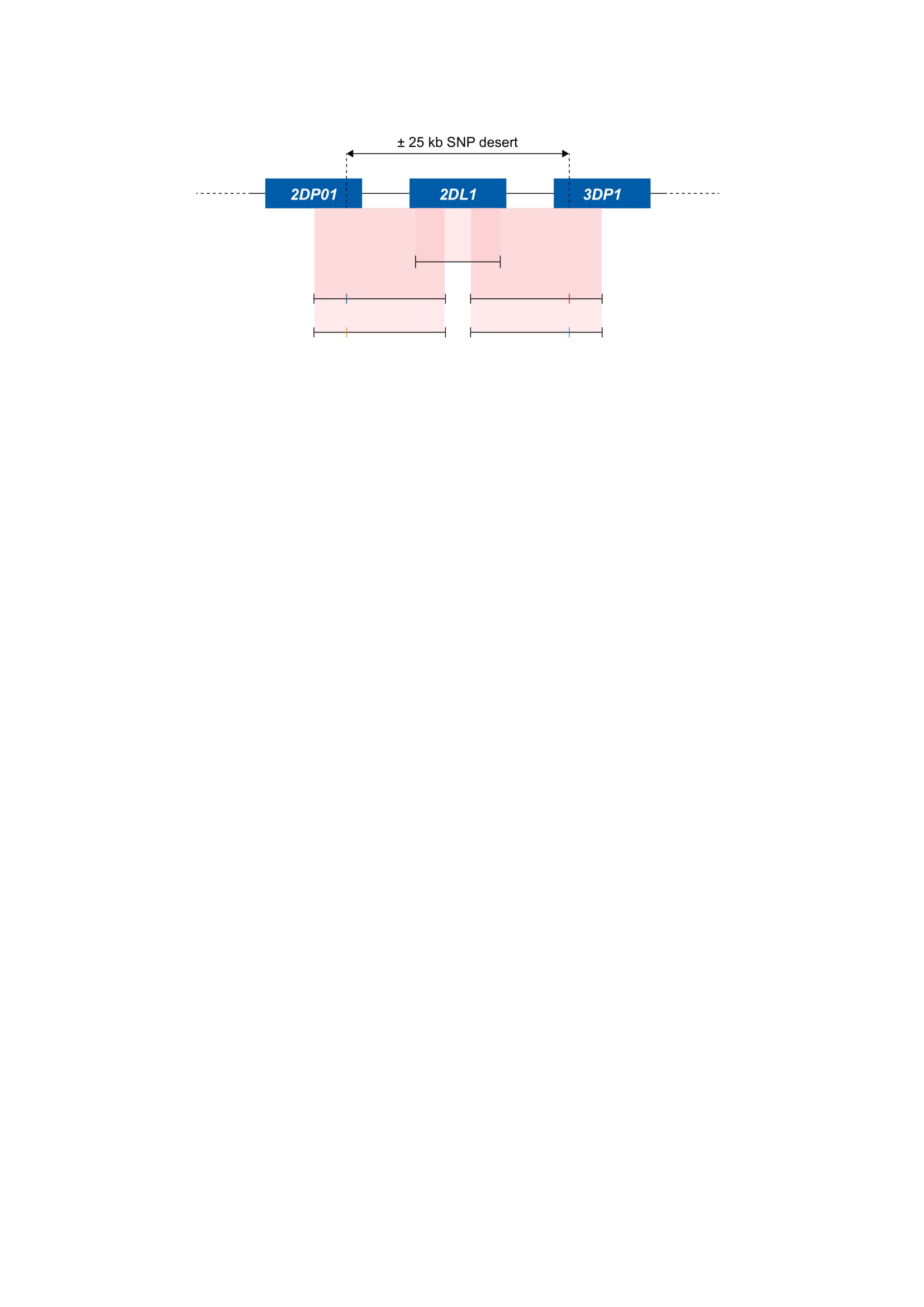

Supplement: Supplementary Figure 1 — A SNP desert at the KIR2DL1 gene. Human individual #3 shared an identical KIR2DL1 allele at both haplotypes, which located a 25 kb SNP desert. The lack of reads that span the complete SNP desert hampered the phasing of KIR2DL1. [file Image_1.jpeg]
